# “Down the Language Rabbit Hole with Alice”: A Case Study of a Deaf Girl with a Cochlear Implant

**DOI:** 10.1155/2011/326379

**Published:** 2011-10-24

**Authors:** Jean F. Andrews, Vickie Dionne

**Affiliations:** ^1^Department of Deaf Studies and Deaf Education, Lamar University, P.O. Box 10076, Beaumont, TX 77710, USA; ^2^Department of Speech and Hearing Sciences, Lamar University, P.O. Box 10076, Beaumont, TX 77710, USA

## Abstract

Alice, a deaf girl who was implanted after age three years of age was exposed to four weeks of storybook sessions conducted in American Sign Language (ASL) and speech (English). Two research questions were address: (1) how did she use her sign bimodal/bilingualism, codeswitching, and code mixing during reading activities and (2) what sign bilingual code-switching and code-mixing strategies did she use while attending to stories delivered under two treatments: ASL only and speech only. Retelling scores were collected to determine the type and frequency of her codeswitching/codemixing strategies between both languages after Alice was read to a story in ASL and in spoken English. Qualitative descriptive methods were utilized. Teacher, clinician and student transcripts of the reading and retelling sessions were recorded. Results showed Alice frequently used codeswitching and codeswitching strategies while retelling the stories retold under both treatments. Alice increased in her speech production retellings of the stories under both the ASL storyreading and spoken English-only reading of the story. The ASL storyreading did not decrease Alice's retelling scores in spoken English. Professionals are encouraged to consider the benefits of early sign bimodal/bilingualism to enhance the overall speech, language and reading proficiency of deaf children with cochlear implants.

## 1. Introduction

After the pediatrician and the newborn health screener technician, pediatric otolaryngologists and audiologists are parents' first professional contact. Typically, after the diagnosis of hearing loss, they make the referrals to the early education specialists [1]. Training for medical/audiological professionals typically is focused on identification and collection of quantitative data about the child's physiological hearing loss. Most have not studied the impact of deafness on language and emergent reading development or have critically examined the benefits of another communication option for deaf children with cochlear implants (deaf/CI), that of early sign bimodal/bilingualism [1–5]. These areas are typically relegated to the speech-language pathologist, deaf educator, and reading specialist. The purpose of this paper is to cross disciplines to inform medical and audiological professionals about an alternative communication option—sign bimodal/bilingualism for deaf children with cochlear implants. Related to the theme of this special issue, we suggest that pediatric medical/audiological professionals can accentuate the “pearls” or benefits of the implant (i.e., improved speech production) and decrease the “pitfalls,” of the implant. We see one pitfall as the limiting of the deaf/CI childs language and literacy learning potential to one language (monolingualism) instead of broadening their language-learning base with sign bimodal/bilingualism (two languages). 

Indeed, every audiologist already knows that even with Early Hearing Detection Intervention (EHDI) in place, still many deaf newborns are missed. As a result, they often receive late intervention and experience language delays. Moreover, many early identified children with even the best surgical outcomes and professional and parent training may never develop spoken language or emergent literacy skills in the same manner as hearing children do [6–8]. We speculate that sign bimodal/bilingualism may be a viable solution for interested parents for late implanted and even early implanted deaf children because it opens both pathways—visual and auditory—to language learning as early as possible [4, 5].

## 2. Deaf/Cochlear Implant (Deaf/CI) Child as Bimodal/Bilingual

According to Grosjean [9], bilingualism does not necessarily mean full proficiency in two languages. It means that the individual uses both languages for everyday purposes. Indeed, by introducing American Sign Language (ASL) (bilingualism) and sign-supported speech (bimodalism) as another language avenue in addition to spoken English, this may ensure early and full access to language. The advantages of early sign bimodal/bilingualism are that it enriches the deaf child's early capacity to think and communicate with parents, extended family and friends and learn to read and access the school curriculum [10–13]. Another advantage is that signs can enhance the learning of spoken English as deaf children can “piggyback” their speech vocabulary skills onto their sign language vocabulary skills [14, 15]. A disadvantage of bilingualism is that if both languages remain underdeveloped, then the child becomes semilingual or weakly proficient in both languages. Semilingualism has tremendous negative consequences especially if the semilingual deaf youth or deaf adult gets caught in the criminal justice system. They cannot participate in their criminal defense because of their weak sign skills and weak English skills thereby jeopardizing obtaining their constitutional rights of due process [16].

Sign bimodal/bilingual users utilize both code-switching and code-mixing strategies. Code-switching is “moving from one language to another, inside a sentence or across sentences,” [17, page 699]. It refers to the “subtle” and “purposeful” way in which bilinguals switch between the two languages [17, page 37]. In contrast, code-mixing has been defined as “the mixing of two languages within a sentence or across sentences [17, page 699]. Code-switching and code-mixing have also been linked to language contact studies which examine concepts such as language transfer, borrowing, and interference [17, pages 51, 7]. 

We differentiate our communication definition of CS from other CS definitions with adult language users who have full access and proficiency in both languages such as interpreters, teachers, and deaf adults who had deaf parents [3]. While Alice, the child in this case, study had neither full access nor native proficiency in both languages, we still view her as an emerging bimodal/bilingual who wears a cochlear implant and who used both codeswitching and code-mixing strategies in two languages to enrich her communication, literacy, and language learning.

Using Pinker's (NASL signs are glossed in capital letters. Spoken words are shown in lowercase letters. Fingerspelling is shown in hyphenated capital letters and SimCom or codemixing is shown with sign capitalized and voice made at the same time in smaller case letters. For example, CAT: ASL sign, cat: spoken word, C-A-T: fingerspelled word, CAT/cat: (SC) SimCom sign and spoken word at the same time.) [18] application of the rabbit hole metaphor to explore “hidden mental worlds”, we take our readers “down the rabbit hole” to see how Alice develops both of her languages by looking at two data sets: (1) her language assessment profile and (2) her responses to the storytelling experiments. 

## 3. Methodology

In this study, we utilize qualitative descriptive tools used in reading research [19, 20]. These narrative accounts are used by observers to make verbatim accounts of the child's use of language while reading a text or answering questions from the reading teacher [21, 22]. Observers then find trends or patterns in the data to identify thinking and reading processes [19, 22]. The dependent variables are reading and language behaviors that can be described and quantified (i.e., retelling of events in storybook or number of times the student codeswitches and codemixes while using the two languages and the independent variable is the intervention, the use of storybook reading to faciltiate early reading development [22]). 

### 3.1. Intertranscriber Reliability

Transcripts of the spoken and signed conversations of Alice were transcribed into English print with an 80% agreement noted by an independent transcriber.

### 3.2. Research Questions

We addressed the following research questions. (1) How does Alice use her sign bimodal/bilingualism, code-switching, and code-mixing strategies during general camp activities (i.e., play, speech lessons, auditory listening sessions, art, and at lunch time)? (2) What sign bilingual code-switching and code-mixing strategies did she use during reading activities while attending to a story told in sign only and speech only and in retelling wordless picture books when read to her in speech only and in sign only?

### 3.3. Experimental Materials

Wordless picture books and books with repetitive and predictable phrase and sentence patterns were used: *Frog Where are You?* by Mayer [23, 24]. Other stories included *A Boy, A Frog, and a Dog *[24]. Story 3 was* Just Me and My Mom* [25]. We also used the book, *There Was An Old Lady Who Swallowed a Fly,* that had repetitive and predictable sentence patterns.

### 3.4. Setting

The setting of the study was a summer reading camp located at a preprofessional university training institution. Bachelors level, Master's and Doctoral level students in communication sciences, speech-language pathology, audiology and deaf studies/deaf education preparing for careers in working with deaf children with cochlear implants were observers and participants. Our case study, Alice, attended camp for four weeks, four days a week from 9:00 AM to noon. Deaf and hearing staff and visitors ate lunch with the six children in order to promote additional conversation skills.

The reading camp was centered around storybook reading. Mason [22] has conducted a series of intervention studies and has reviewed other studies which show causal links between storybook reading to children and emergent and later reading achievement. Thus, we utilized a deaf native signer to provide the ASL telling and reading of the story and a speech-language pathologist to provide the stories in spoken English only (see [26] for similar procedures). Following up activities included discussion of concepts and vocabulary, relating story to students' personal experiences, art and play-acting experiences using both languages. Upon this rich language base, children in groups or individually met with the speech-language pathologist or audiologist to listen to stories told in oral language and to build spoken and auditory language skills. Just as Yoshinago-Itano [14] “piggy-backed” speech onto sign at the word level for deaf/CI infants, we aimed to investigate the effects of “piggy-backing” spoken English stories onto stories told/read using American Sign Language for purposes of examining code-switching and code-mixing behaviors.

## 4. Results

Alice, a Caucasian girl, was born deaf from unknown causes. At age three, she began an early intervention program in a deaf regional day school and she started to learn sign language provided by a hearing parent-infant educator. Her primary caretaker is her grandmother who knows little signs and has a high school diploma. Alice is in the second grade in a self-contained deaf education classroom. Her teachers use sign-supported speech and ASL with her.

Alice scored a 118 IQ on the TONI-3 which is above the average range. On Wesby's Play and Theory of Mind Scale (adapted from [27]), the team evaluators placed her at Level IX (age 3.5 to 4 years) which observes play activities in the camp. Related to corresponding language behaviors at this level, she used conjunctions (and) and began to respond to why and how questions that require reasoning about perception. These language behaviors were observed in American Sign Language (ASL), spoken English, and in a mixture of signs and speech.

### 4.1. Language Assessments

#### 4.1.1. Audiology, Listening, and Speech Perception

The speech-language pathologists and audiologists could not test Alice for speech reception without visual cues. Alice's score of 37 dB hearing loss refers to the sound field pure tone average with her implant. At the 500 Hz, Alice scored 40 dB; at the 1000 Hz, she scored 35 dB; at the 2000 Hz, she scored 30; at the 4000 Hz, she scored 30. It is important to note that the degree of hearing loss does not equate in a linear way with the child's ability to hear conversational speech and comprehend language in real-world environments such as noisy classrooms, on a playground, or in the school cafeteria [2, 3]. The degree of hearing loss expressed in decibels may show impressive decreases from 100 dB without the implant to 37 dB with the implant. However, such an average inaccurately predicts the child's functional hearing in noisy real-world environments or the ability to comprehend and produce language through audition alone.

#### 4.1.2. Speech Perception

Prior to age 3.5 when she was implanted, Alice was nonverbal and only used signs for communication. With the implant and training, she did make marginal gains in speech production. However, her speech was for the large part unintelligible. Alice was administered the SD/PBK, a *Speech Discrimination Testing Using the Phonetically Balanced Kindergarten Word Lists* [28]. Alice was unable to do this test. Alice was also administered the WIPI, the Word Intelligibility by Picture Identification Test (WIPI) [29]. She scored 56% out of 25 possible points, without visual cues. She was also given the six-sound Ling test, and without visual cues, she was able to recognize the sounds: ah, ee, sh, s, and m with 100 percent accuracy. She recognized the oo sound with 40% accuracy.

#### 4.1.3. Receptive Spoken Language Test

Alice was administered the Peabody Picture Vocabulary Test (PVVT), 4th edition [30]. The PPVT is a single-word vocabulary test where the child views the four picture plates and identifies the word the test administrator says. She scored in the 4-year-old range for receptive spoken vocabulary, 3.5 years behind her chronological age of 7 years, 5 months.

#### 4.1.4. Use of Auditory Technology

Alice was implanted at the age of four years and four months. She had the implant for three years and one month. She had not used a hearing aid prior to implantation. She was fitted with an implant made by Cochlear Corporation with a Nucleus Freedom BTE speech processor. She was mapped 10 months prior to the reading camp at a hospital in a larger metropolitan city, 80 miles away.

#### 4.1.5. Reading and Writing (Literacy)

When reading words and paragraphs on the reading assessments, Alice translated the printed English words into signing as she codeswitched from English to signing. When she did not know a work in a text, she would fingerspell it. Alice was tested to be reading at the first-grade level. She was given an informal reading inventory [31] and scored at the first-grade instructional level, Kindergarten independent level- and 2nd-grade frustration level.

When reading a story about a pet dog on the IRI, Alice would sign word for word in the paragraph. While she could match printed words in the sentence with manual signs with accuracy and facility, when she was asked questions about the story as a whole, she could not answer them. Thus, she was able to code-switch from print to sign at the word level, but she could not integrate her understanding of the specific word into the context of the sentences or the complete story. 

She was administered the Test of Early Reading-3 for Deaf and Hard of Hearing (quotient 109, 75 percentile rank) on a test that measures alphabet knowledge, conventions, and meanings of print. On the TERRA-3, she could recognize about 20 sight words. When asked to decode or figure out the meaning of a printed word, she did not use any phonemic awareness or phonics, but relied on visual strategies. Alice made miscues or mistakes in reading based on letter visual similarities such as identifying the sign BROWN for the word, *down*, LONG for *log*, and FRIEND for *then*.

We presented Alice with a list of words and we asked her to sound out the words, but she could not do this task. Based on her audiogram denoting a 37 dB loss with her implant, we thought she could use sounding-out strategies for word decoding, but she could not phonologically decode familiar words, unfamiliar words, or pronounce nonwords. Related to her expressive writing behaviors, Alice could label her drawings with single words. She was not able to write complete sentences unless she copied them from the whiteboard model during our reading camp activities.

### 4.2. Sign-Supported Speech, ASL, and Fingerspelling

We determined that Alice knew the fingerspelled alphabet and could spell short three- to four-letter words using fingerspelling. We also observed Alice using sign-supported speech with hearing adults and ASL with the deaf adults at our summer reading camp. We also administered to Alice the *Carolina Picture Vocabulary Test* (CPVT). The CPVT contains many signed English signs, so it does not measure ASL exclusively. Alice scored above her age range on this test. The native deaf evaluators on our staff administered to Alice the Kendall Conversational Proficiency Level Assessment [32]. This is a rating scale that assessed Alice's sign language communication competency across three levels from 0 to level 7. Alice obtained a level 3 conversational proficiency level. Alice was able to converse in sign language about topics which interested her, refer to a number of objects and actions, and communicated about the location of objects. She could discuss characteristics of people and objects, communicate about what she owns, link her conversations to others, and use sign to affirm the presence of objects, identify pictures using signs, and use at least two syntactically related components in sign language.

### 4.3. Research Question Number 1: How Did Alice Use Her Sign Bimodal/Bilingual Code-Switching and Code-Mixing Strategies During General Camp Activities?

Our videotaping data, field notes, and notes from her files revealed that Alice used code-switching or code-mixing from one language to another (ASL to speech, ASL to print, and sign-supported speech to print) with different communication partners, in various settings, and for various reasons. For the most part, she used speech and sign-supported speech with their hearing parents, siblings, and hearing staff members of the camp that could not sign. But, she utilized code-mixing with deaf native signers as well. 

When walking across campus on a field trip, she would often stop at environmental signage and Alice would stop and attempt to read the print. When she could not identify any words, they would fingerspell the words. She did the same when she was looking at print in books; when they came across a word they did not know, they would fingerspell the word. During therapy time with the audiologist and the speech pathologist, she would code-switch to speech or sign supported-speech. On the playground, the Alice liked to use sidewalk chalk to print their names and draw pictures and label them with letters and words. She would use ASL to explain these pictures and print words to her teachers and classmates.

On the playground and at lunch, Alice would code-switch from ASL to speech when using social words such as *please, thank you or sorry, or excuse me*, when they were in emotional or physical pain (i.e., falling down on the playground) or expression of surprise (uh-oh, oh, no) and to use emotion words such as *sorry* and *I love you*. She would code-switch to speech to emphasize, repeat, or reinforce a point or express a strongly felt attitude in a story, and yell out loud, *yes* or *no *and to imitate hearing people using cell phones or singing into a microphone. She would code-switch from ASL conversation to fingerspelling when coming across a word she did not know in a storybook or on environmental signage around campus, when labeling pictures in a book or retelling a story. She would codemix ASL and sign-supported speech when telling something that happened at home or on a family outing. She would codes-witch from ASL or sign-supported speech to fingerspelling when using a name sign to get the attention of a staff or peer member of the camp, when working with the SLP or the audiologist who did not knew little or no sign, or when communicating with the native deaf storytellers at camp and the deaf graduate students who joined them for lunch. When attending to a story from the native deaf storyteller, she would answer questions in ASL and when asked to retell segments of stories. She would code-switch to sign-supported speech when reading word for word in a storybook.

### 4.4. Research Question Number 2: What Sign Bimodal/Bilingual Code-Switching and Code-Mixing Strategies Did She Use During Reading Activities? (A) Attending to a Story Told in Sign Only and Speech Only. (B) Retelling Wordless Picture Books When Read to Her in Speech Only and in Sign Only

The first session was presented in ASL by a native deaf signer, then the next story was presented in spoken English by the speech-language pathologist. The story used was *There Was an Old Woman Who Swallowed a Fly*. 

Todd, the native deaf signer, signed the whole story in ASL pointing to the pictures as he goes along. Here are some excerpts from Todd's ASL storytelling with students' responses (Box 1).

In Box 2, two speech-language pathologists, Ann and Faye, tell this same story in spoken English only.

### 4.5. Discussion

In the ASL storytelling rendition, two of the camp children—Bailey and Alice—used speech and sign (codemix) to answer a question and to label a picture (SPIDER/spider CRAWL). Bailey uses code-mixing to emphasize a point (CATCH/catch). Alice and Bailey code-switch to speech (no) to express disgust at a woman eating the spider. Alice switches to speech to make the animal sounds of a cat (meow, meow). Bailey codeswitches to speech to excitedly answer Todd's predictive question of what lies ahead (dog!). Bailey codeswitches to speech to make the sound of a dog (woof, woof). Alice switches to speech to Todd's ASL classifier (puffed cheeks) and says fat. Alice uses code-mixing to answer a question (cow). Then, Alice sees Bailey's sign for COW. Alice uses speech to say cow. Alice answers Todd's question and says horsey.

In the spoken-language story rendition, the two girls principally used speech to label pictures of animals, to label actions, to label sounds that animals and birds made, and to show enjoyment of the story (vocalizations during laughter) or to express negation (no). After the spoken repetition of the line in the story, “I do not know why she swallowed the fly!” said by one of the hearing story readers (Ann), one of the girls (Alice) said in a complete sentence: I do not know why. The girls also used signs to support their speech, either using signs and speech simultaneously or separately. (Alice: BUGS/bugs), (BAILEY: BIRD/bird).

Todd utilizes a number of strategies similar to those found with deaf parents reading to their deaf children [26]. He used signing to build background knowledge, to share personal experience tied to book theme, and to persons and objects in pictures. He also asked questions using signing to check the children's comprehension. Not using fingerspelling extensively, he did fingerspell a central word in the book—F-L-Y—for the general sign of BUG, INSECT. He frequently paused and waited for reply, He maintains visual attention and recovered attention when the children became visually distracted. He play-acts the story. He assumed the role of the characters, modeling their emotions. He modeled individual signs, classifiers, sign sentences so that children can imitate. He encourages turn-taking and gives children's time to respond. He repeats ideas in the book using signs and made comments on the story. Todd did not use code-mixing in his ASL storytelling. He did use some code-switching from sign to fingerspelling and while pointing at print and giving the sign language equivalent. But for the most part, he used ASL.

Having laid the concepts for the story using ASL by Todd, Faye and Ann presented the same story in spoken language. Similar to Todd, Faye and Ann used a number of strategies using speech to make the storytelling enjoyable and entertaining for the children as well as to teach them speech skills. Faye used signs (SimCom) to support the children's learning of speech. Both clinicians used speech to help the children label pictures in books, to support children's learning of words, to ask questions, to praise, to model the speaking of complete sentences, to redirect questions, and to encourage turn-taking. Faye used speech to ask questions about the characters in the book. Ann used speech to set up contrasts of concepts for spoken words (old versus young). They used speech to teach the sounds that animals and insects make. They used speech to praise the child's response. They also used speech to redirect an incorrect answer to questions.

The children used code-switching and code-mixing with their storytellers to emphasize a point. They used speech to express emotion such as excitement or disgust and to enhance their storytelling by making the sounds of animals. The girls used code-mixing to support their use of speech and their use of ASL. Alice would view an ASL classifier (puffed cheeks), then use the spoken word, fat. During the spoken story, Alice uses speech to label pictures and to answer questions.

## 5. Retelling Wordless Picture Books

In the next experiment, Alice was read four wordless picture books and predictable books in ASL and in spoken language only.  Table 1 shows the data on the number of incidences where she used ASL only and speech only in her retellings of the four stories. The table also shows the number of times she codeswitched and codemixed during her retellings of each story.

Regarding Alice's expressive speech language, her speech utterances numbered 11 after the ASL rendition of the first story and increased to 23 after the spoken English rendition of the new story. Alice made 24 and 22 codemixes after the stories were told in ASL and decreased to only 3 codemixes with the last story being told in speech only. The ASL utterances remained low for both after the ASL storytelling and the spoken English storytelling.

## 6. Development of Code-Switching and Code-Mixing Scale Based on Developmental Data of Six Deaf/CI Children

From Alice's data and from the other five deaf/CI children in the camp, we developed a four-point scale filled out by three hearing and three deaf evaluators who worked with the children in the camp. They collectively placed Alice at level three (Box 3).

From our observations, Alice was at level 3 of this scale. She codeswitched at the phrase level.

## 7. Discussion

If we climb down “the rabbit hole” with Alice and observe her language and reading processes, we see her sign bimodal/bilingualism has rich, expressive power. Viewed thusly, we can get a more complete picture of Alice's language and literacy-learning potential in both languages. We, capitalizing on the “pearls” or benefits of cochlear, implant technology (improved speech production) and avoid one “pitfall” of being limited to one language-learning pathway. We speculate that sign bimodal/bilingualism through parent and teacher storybook reading may even benefit those deaf infants who are identified early and receive early intervention because it opens both pathways to language learning and enhances the cognitive, language, emergent literacy, and psycho-socioemotional capabilities of deaf children [4, 5]. More interdisciplinary research conducted by teams of early childhood educators, speech-language pathologists, educational audiologists, deaf educators, and reading specialists are needed to substantiate this speculation. Such research would enhance the potential of pediatric otolaryngologists and audiologists in providing information about communication and language options for families of deaf children with cochlear implants.

## Figures and Tables

**Box 1:**
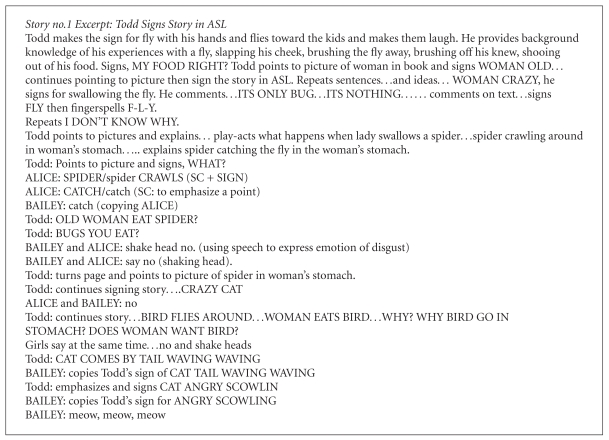


**Box 2:**
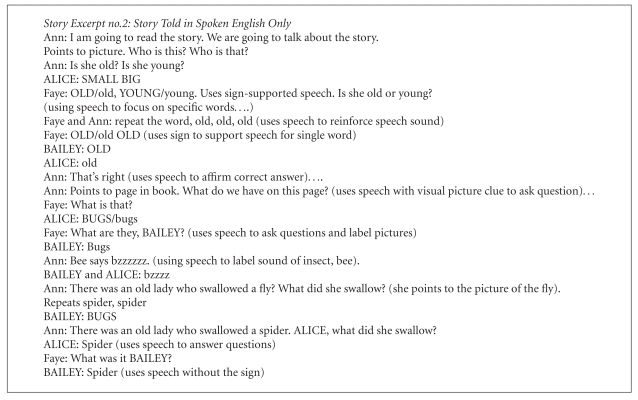


**Box 3:**
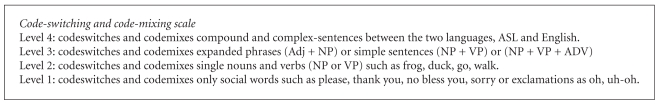


**Table 1:** Summary of Language Use by Alice in Four wordless book storybook retellings.

Stories	Uses ASL only	Uses speech only	Uses codeswitches/codemixes
ASL Story (Frog Where Are You?)	1	11	24
Spoken English (Frog Where Are You?)	2	25	13
ASL Story (A Frog, A Dog, A Boy)	4	5	22
Spoken English (Just Me and My Mom)	1	23	3
